# Association between vitamin D status and eryptosis–results from the German National Cohort Study

**DOI:** 10.1007/s00277-023-05239-w

**Published:** 2023-05-01

**Authors:** Franz Ewendt, Marvin Schmitt, Alexander Kluttig, Julia Kühn, Frank Hirche, Frank B. Kraus, Beatrice Ludwig-Kraus, Rafael Mikolajczyk, Wim Wätjen, Paul-Christian Bürkner, Michael Föller, Gabriele I. Stangl

**Affiliations:** 1 Institute of Agricultural and Nutritional Sciences, Martin Luther University Halle-Wittenberg, 06120 Halle (Saale), Germany; 2 Cluster of Excellence SimTech, University of Stuttgart, 70569 Stuttgart, Germany; 3 Institute of Medical Epidemiology, Biostatistics, and Informatics, Medical Faculty of the Martin Luther University Halle-Wittenberg, 06120 Halle (Saale), Germany; 4 Central Laboratory, Department of Laboratory Medicine, University Hospital Halle, 06120 Halle (Saale), Germany; 5 Department of Physiology, University of Hohenheim, 70599 Stuttgart, Germany

**Keywords:** Apoptosis, Red blood cells, 25-Hydroxyvitamin D, Anemia, Cardiovascular risk

## Abstract

Vitamin D, besides its classical effect on mineral homeostasis and bone remodeling, can also modulate apoptosis. A special form of apoptosis termed eryptosis appears in erythrocytes. Eryptosis is characterized by cell shrinkage, membrane blebbing, and cell membrane phospholipid disorganization and associated with diseases such as sepsis, malaria or iron deficiency, and impaired microcirculation. To our knowledge, this is the first study that linked vitamin D with eryptosis in humans. This exploratory cross-sectional trial investigated the association between the vitamin D status assessed by the concentration of plasma 25-hydroxyvitamin D (25(OH)D) and eryptosis. Plasma 25(OH)D was analyzed by LC–MS/MS, and eryptosis was estimated from annexin V-FITC-binding erythrocytes by FACS analysis in 2074 blood samples from participants of the German National Cohort Study. We observed a weak but clear correlation between low vitamin D status and increased eryptosis (*r* =  − 0.15; 95% CI [− 0.19, − 0.10]). There were no differences in plasma concentrations of 25(OH)D and eryptosis between male and female subjects. This finding raises questions of the importance of vitamin D status for eryptosis in terms of increased risk for anemia or cardiovascular events.

## Introduction 

Vitamin D in its active form 1,25(OH)_2_D_3_ (calcitriol) plays a decisive role in the regulation of mineral homeostasis and bone remodeling [1]. In the last two decades, many other cellular and physiological processes such as proliferation, differentiation, survival, maturation, and activation of cells have been linked to 1,25(OH)_2_D_3_ [2–4]. With the discovery of extra-skeletal vitamin D effects, studies have emerged to correlate vitamin D status with cardiovascular risk factors, autoimmune diseases, COVID-19, other respiratory infections, or neurological disorders [5–7].

By comparison, relatively little is known about the effect of vitamin D on the hematologic system, although several observational studies found that a low vitamin D status is associated with a higher anemia risk in healthy subjects and patients with chronic kidney disease [8–12]. The proposed underlying mechanisms comprise the increase in proinflammatory cytokines and iron-regulatory hepcidin in situations of insufficient vitamin D levels which lead to iron sequestration and reduced dietary iron absorption [13]. Additionally, clinical observations assume a possible role of vitamin D in erythropoiesis [14] because hemodialysis patients who received a vitamin D hormone analogue needed fewer erythrocyte-stimulating agents and had more reticulocytes in the blood than those receiving no analogue [15]. These data suggest a protective effect of vitamin D on the development of anemia. In contrast, mice placed on a high-vitamin D diet for 2 weeks had lower plasma erythropoietin levels and a lower mean corpuscular volume than mice fed a control diet [16].

Similar to apoptosis of nucleated cells, erythrocytes can undergo a certain type of programmed cell death, which is referred to as eryptosis [17]. Eryptosis includes cell shrinkage and cell membrane scrambling and is triggered by factors that induce the cellular influx of Ca^2+^ ions. Intracellular Ca^2+^ promotes the translocation of phosphatidylserine (PS) from the inner to the outer cell membrane [17]. PS-exposing red blood cells are rapidly phagocytosed, but can also adhere to the inner blood vessel walls and stimulate blood clotting, which may lead to a dysfunctional microcirculation and an increased risk for cardiovascular events [18]. In mice fed a high vitamin D diet for 2 weeks, it was observed that eryptosis tends to be lower than in control mice [16]. This study in mice was the first that linked vitamin D to eryptosis.

Vitamin D deficiency and insufficiency are highly prevalent in European populations [19, 20]. Hence, there are recommendations for vulnerable groups to take vitamin D supplements. Whether vitamin D status is linked to eryptosis in humans is currently unknown. The current exploratory cross-sectional study aimed to investigate plasma levels of 25-hydroxyvitamin D (25(OH)D) as a biomarker of vitamin D status and eryptosis in more than 2000 individuals from the German National Cohort (NAKO) Study. To our knowledge, this is the first study addressing vitamin D status and eryptosis in humans, and the first time that eryptosis was measured in a larger population group. It is important with respect to the fact that vitamin D deficiency and diseases caused by dysfunctional microcirculation are widespread and a relevant health issue. Our study may also contribute to the assessment of the role of vitamin D in disease-related anemic conditions that may be aggravated by accelerated yet occult eryptosis.

## Methods

### Population and study design

To investigate the proposed association between vitamin D status and eryptosis, blood samples from 2074 participants of the NAKO in the study center in Halle (Saale) were analyzed for 25(OH)D and eryptosis. The study design of the NAKO has been described in detail elsewhere [21–24]. In brief, the NAKO is a population-based prospective study that includes more than 205,000 participants between 20 and 69 years of age at baseline from 18 study centers across Germany. The overarching goal of the NAKO study is to identify risk factors and exposures including socio-economic, psychosocial, lifestyle, and environmental factors that contribute to common diseases such as cardiovascular diseases, diabetes, cancer, and neuropsychiatric, infectious, and musculoskeletal disorders. Baseline examinations included self-administered questionnaires and interviews on health status, lifestyle, and anthropometric measurements [24]. Data collection in the NAKO follows standardized operating procedures, and internal and external quality control is conducted.

### Ethics statement

The current study was approved by the ethical review committee of the study center at MLU Halle-Wittenberg (L3 Project: Processing number: 2013–22). Written informed consent was obtained from all participants.

### Recruitment and data collection

Participants in this study were recruited during the regular first follow-up of NAKO participants from 04/10/2019 to 11/04/2021. During the informed consent process for NAKO, participants were asked whether they would be willing to participate in our subproject and had an additional 3-ml plasma tube drawn. Of a total of 3190 NAKO study participants examined during the study period mentioned above, an additional blood sample was obtained from 2174 individuals.

Data utilized in this study included age, sex, smoking, body mass index (BMI), hematological parameters (reticulocyte and erythrocyte count, hematocrit and hemoglobin), and 25(OH)D. Blood samples were collected from Monday to Thursday in the Halle study center, and then transferred to the Institute of Agricultural and Nutritional Sciences, where the blood samples were analyzed for their plasma level of 25(OH)D and eryptosis.

### Analytical methods

#### Quantification of plasma 25(OH)D

25(OH)D_3_ and 25(OH)D_2_ were assayed in plasma from Li-heparin blood by using a MassChrome® kit (Chromsystems Instruments & Chemicals GmbH, Gräfelfing, Germany) and high-performance liquid chromatography system (1260 Series, Agilent Technologies, Waldbronn, Germany) coupled to tandem mass spectrometry (QTRAP 5500, Sciex, Darmstadt, Germany). Sample preparation procedures and analytical conditions were done in accordance with the manufacturer’s protocol. The coefficients of variation for 25(OH)D_3_ and 25(OH)D_2_ were 5.1% at 38.6 nmol/l and 5.8% at 40.8 nmol/l, as well as 6.2% at 86.6 nmol/l and 6.4% at 94.0 nmol/l. The lower limit of quantification (LLOQ) was 5.0 nmol/l for 25(OH)D_3_ and 2.43 nmol/l for 25(OH)D_2_. Overall, all 2074 samples had quantifiable concentrations of 25(OH)D_3_, and 1561 out of 2074 samples had 25(OH)D_2_ levels that were below the LLOQ. 25(OH)D levels (nmol/l) were defined as the sum of 25(OH)D_3_ and 25(OH)D_2_.

#### Analysis of eryptosis

Eryptosis was analyzed in fresh blood samples (maximum 24 h old) in duplicate by assessing phosphatidylserine exposure of erythrocytes as published [25]. In brief, to isolate the erythrocytes, an aliquot of 5 µl blood was added to 200 µl Ringer solution (pH 7.4; containing 125 NaCl mM, 5 mM KCl, 1 mM MgSO_4_, 32 mM HEPES, 5 mM glucose, and 1 mM CaCl_2_). After centrifugation at 1800 g for 5 min, the supernatant was removed, and the washing step repeated twice. Erythrocytes were then resuspended and stained with annexin V-FITC (BD Biosciences, Franklin Lakes, NJ, USA) at a 1:500 dilution in 250 µl annexin V-FITC buffer (Ringer solution with 5 mM CaCl_2_) for 20 min at room temperature protected from light. Ten µl of resuspended erythrocytes were analyzed by flow cytometry (Cytoflex, Beckman Coulter, Brea, CA, USA), and the annexin V-FITC-fluorescence intensity was determined at an excitation wavelength of 488 nm and an emission wavelength of 530 nm (coefficient of variation < 1%). Eryptosis (%) expresses the percentage of annexin V-FITC-binding cells of the gated erythrocyte population.

#### Analysis of reticulocytes

To determine the number of reticulocytes in the blood samples, 1.5 µl of whole blood was added to 300 µl Retic-COUNT™ (Thiazole orange) reagent (BD Biosciences, USA) in a 96-well plate in duplicate or to 300 µl of Ringer solution to determine cell autofluorescence. Samples were mixed and stained for 30 min at room temperature protected from light. Flow cytometry (Cytoflex, Beckman Coulter, USA) was performed according to the manufacture’s protocol. The reticulocyte count was expressed as the percentage of Retic-COUNT™-positive reticulocytes of the total gated erythrocyte population.

#### Measurement of hematological parameters

Hematological parameters were measured in the central laboratory of the University Hospital Halle (Saale) from whole blood samples using a Sysmex XN-9000 hematological analyzer according to the manufacturer’s instructions and manuals, with routine maintenance and internal and external quality control procedures.

### Statistical analysis

All analyses were performed with the *R* programming language for statistical computing [26], using the brms package [27] as an interface to Stan [28] for Bayesian inference. All Bayesian analyses used weakly informative priors, since prior information was not available due to the exploratory nature of this study. Analyses consistently used four MCMC chains, each with 2000 draws from the posterior distribution (after 1000 warmup samples), and each model was checked with appropriate diagnostics [28]. If not specified otherwise, results are reported as Bayesian posterior means with 95% credible intervals (CI). Mean comparisons are based on models without equal variance assumption, i.e., modeling separate variances per group. In the description of the results, *r* denotes the Pearson correlation coefficient and *d* denotes Cohen’s measure of effect size for mean comparisons. The software code for all analyses is available in the public repository at http://www.github.com/marvinschmitt/nako-eryptosis under GPLv3 license.

### Data pre-processing protocol

Of the 2174 blood samples obtained, cases were dropped if at least one of the following phenomena was observed: hemolysis of the blood sample, coagulation of the blood sample, insufficient storage, or insufficient sample volume. After exclusion, a total of 2100 samples remained. Cases with extreme values (median ± 3 inter-quartile-range) of eryptosis or 25(OH)D_3_ (24 cases) as well as cases with invalid age entries (2 cases) were omitted, leading to the total sample size of 2074. Eryptosis was measured twice, and the arithmetic mean was used in the analyses if both measurements were available. 25(OH)D_2_ levels below the LLOQ (1561 cases) were replaced by values sampled from the uniform distribution between 0 and the LLOQ, to avoid bias, error rates, and reduced variance which would result from a replacement of the missing values by zero or LLOQ/2 [29].

In accordance with established recommendations [30], we categorized the 25(OH)D levels of the subjects into the following groups: deficient (≤ 30 nmol/l), insufficient (30 to < 50 nmol/l), and adequate (≥ 50 nmol/l).

## Results

### Characteristics of the study participants

The study included data from 2074 subjects (977 males, 1097 females). Descriptive statistics of age, BMI, 25(OH)D, eryptosis, reticulocytes, and other hematological parameters are depicted in Table 1. The BMI was available from 2061 participants (821 normal weight, 765 overweight, 475 obese). While 987 participants were non-smokers, 416 subjects are currently smokers, and 602 are former smokers. Due to logistic impairments during the COVID-19 pandemic, the reticulocyte count could be determined only in 2033 participants and the hematological parameters were only available from 2045 (erythrocyte, hemoglobin) and 2044 (hematocrit) participants.

**Table 1:** Characteristics of the study participants

	*n*	Mean	SD	Median	IQR	Min	Max
Age, years	2074	56.44	12.62	57.23	18.21	25.35	77.22
BMI, kg/m^2^	2061	26.94	5.09	26.10	6.10	16.50	61.80
Plasma 25(OH)D, nmol/l	2074	61.84	24.69	59.75	33.06	10.92	158.61
Eryptosis_1_, %	2074	0.97	0.67	0.77	0.57	0.16	5.99
Reticulocyte count_2_, %	2033	1.15	0.65	1.05	0.79	0.00	5.14
Erythrocyte count, Tpt/l	2045	4.58	0.42	4.55	0.56	2.99	6.33
Hematocrit, l/l	2044	0.41	0.03	0.41	0.04	0.32	0.57
Hemoglobin, mmol/l	2045	8.57	0.76	8.50	1.10	6.40	11.60
Leukocyte count, per mm^3^	2035	6.42	1.71	6.19	2.16	2.69	14.09
Thrombocyte count, per mm^3^	2044	253.24	55.39	249.00	70.00	46.00	526.00

_1_% annexin V-FITC-binding erythrocytes; _2_% Retic-COUNT™-positive reticulocytes of the total gated erythrocyte population; *Tpt*, teraparticle

The plasma 25(OH)D of the study participants (Table 1) was deficient in 209 cases, insufficient for 560 subjects, and adequate for 1305 participants, respectively.

### Correlation between plasma 25(OH)D and eryptosis by sex, age, and BMI

**Fig. 1:**
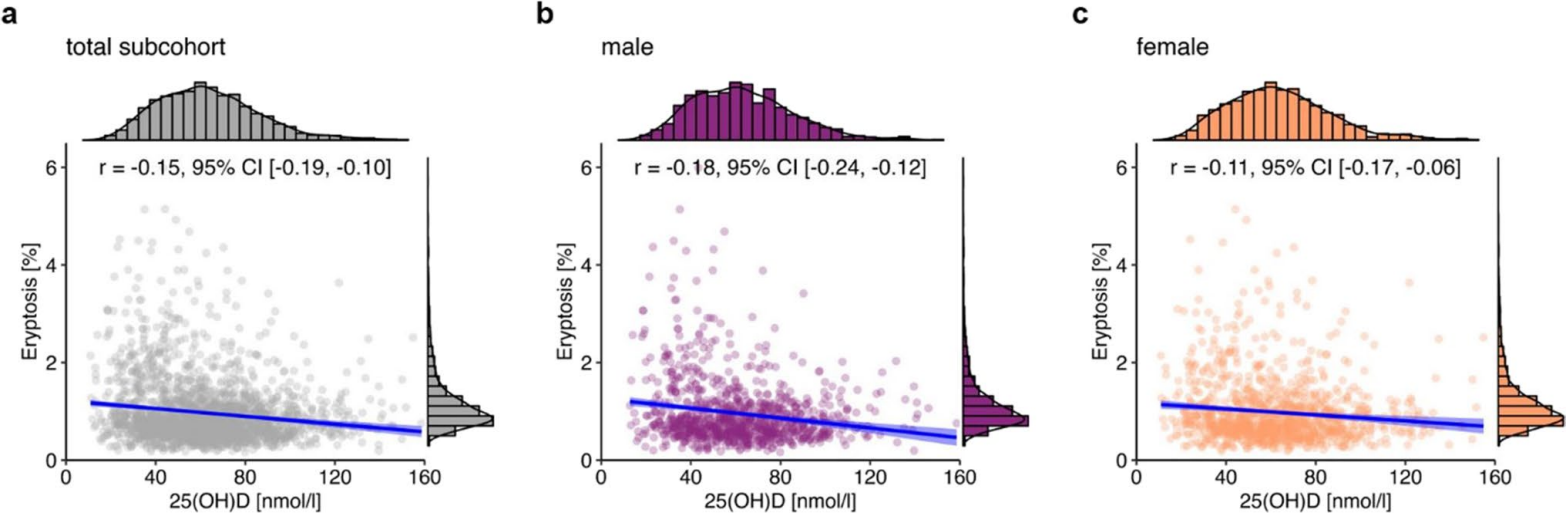
Relationship between 25(OH)D and eryptosis in the total NAKO subcohort (*n* = 2074; **a**), and grouped by sex (**b**: 977 males; **c**: 1097 females). Histograms show the marginal distribution of the respective variable. Lines indicate linear regression, and the blue ribbon around the regression line shows the uncertainty as 95% CI. The figure was created using the *R* programming language

Cross-sectional analysis showed a weak negative correlation between plasma 25(OH)D and eryptosis (*r* =  − 0.15, 95% CI [− 0.19, − 0.10]; Fig. 1a). Between male (Fig. 1b) and female (Fig. 1c) subjects, there were no differences in the plasma concentrations of 25(OH)D (posterior mean difference 0.77 nmol/l, 95% CI [− 1.39, 2.76 nmol/l]) and eryptosis (posterior mean difference 0.03%, 95% CI [− 0.03, 0.08%]).

**Fig. 2:**
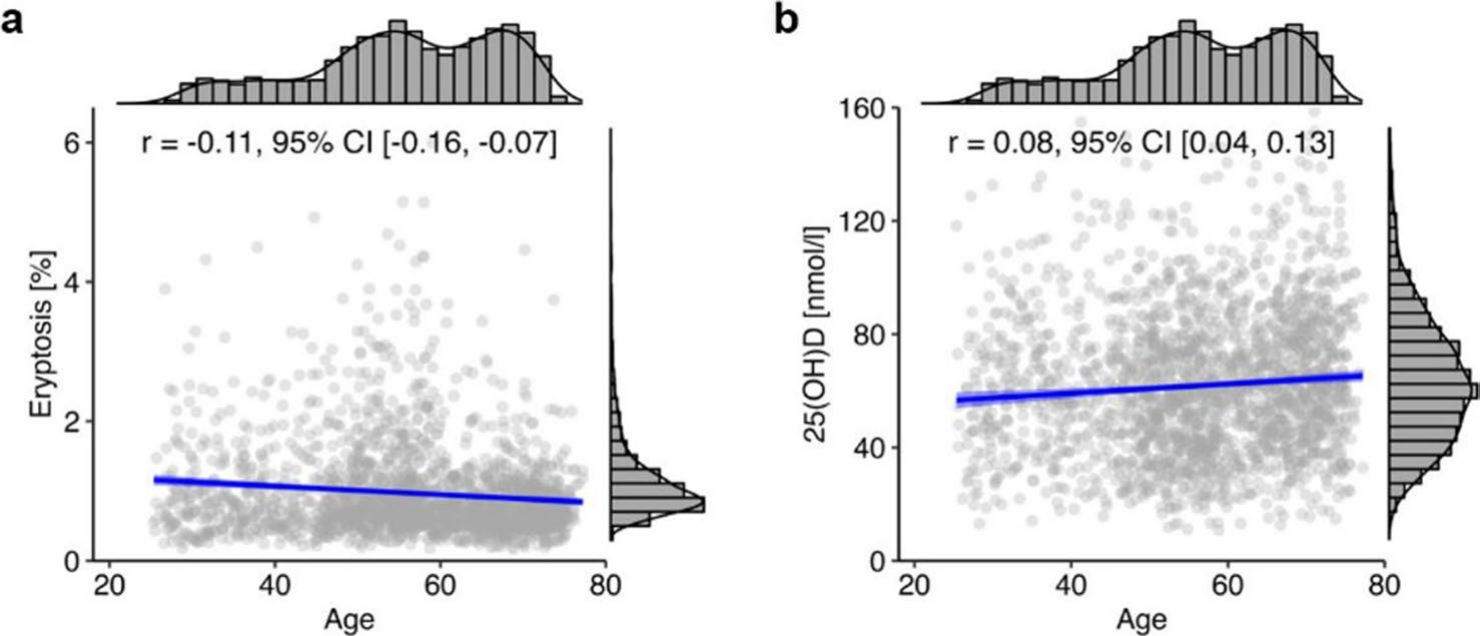
Correlation of age with eryptosis (**a**) and 25(OH)D (**b**) in the total NAKO subcohort (*n* = 2074). Histograms show the marginal distribution of the respective variable, lines indicate regression, and the blue ribbon around the regression line shows the uncertainty as 95% CI. The figure was created using the *R* programming language

Age exhibited a negative correlation with eryptosis (*r* =  − 0.11, 95% CI [− 0.16, − 0.07]; Fig. 2a), and a positive correlation with plasma level of 25(OH)D (*r* = 0.08, 95% CI [0.04, 0.13]; Fig. 2b).

**Fig. 3:**
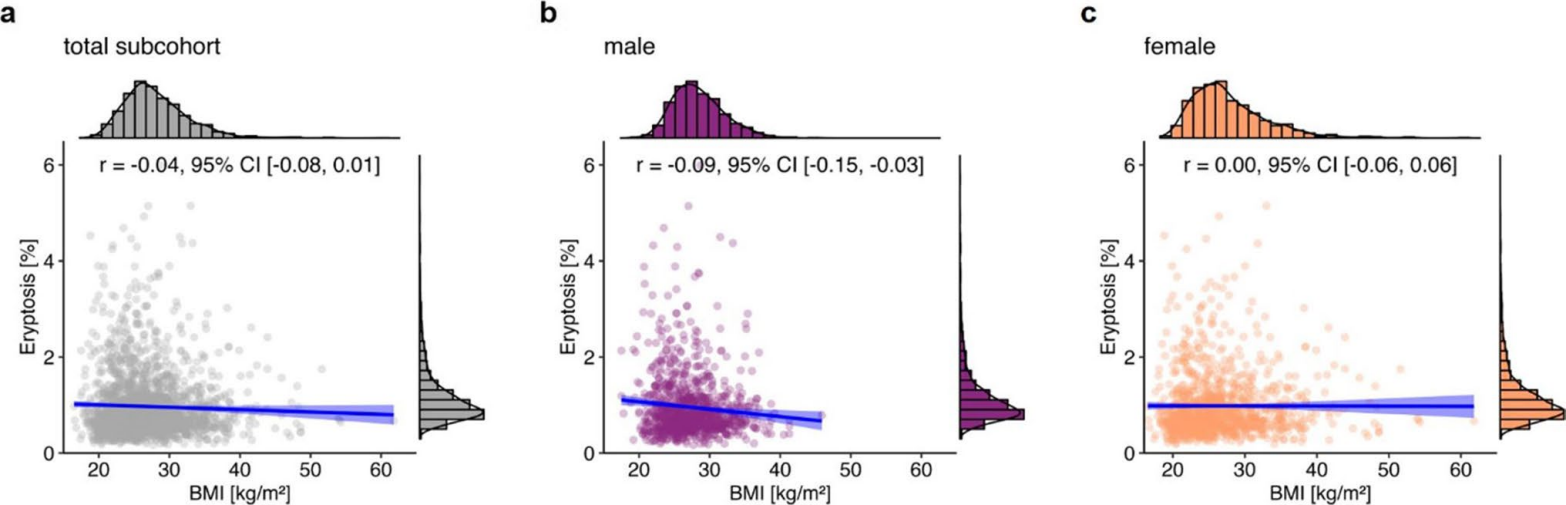
Correlation between BMI and eryptosis in the total NAKO subcohort (*n* = 2061; **a**), and grouped by sex (**b**: 974 males; **c**: 1087 females). Histograms show the marginal distribution of the respective variable, lines indicate regression, and the blue ribbon around the regression line shows the uncertainty as 95% CI. The figure was created using the *R* programming language

Cross-sectional correlation analysis did not reveal a correlation between BMI and eryptosis (*r* =  − 0.04, 95% CI [− 0.08, 0.01]) for the total subcohort (Fig. 3a). However, BMI and eryptosis were more strongly correlated in males (*r* =  − 0.09, 95% CI [− 0.15, − 0.03]; Fig. 3b), than in females (*r* =  − 0.0003, 95% CI [− 0.06, 0.06]; Fig. 3c).

**Fig. 4:**
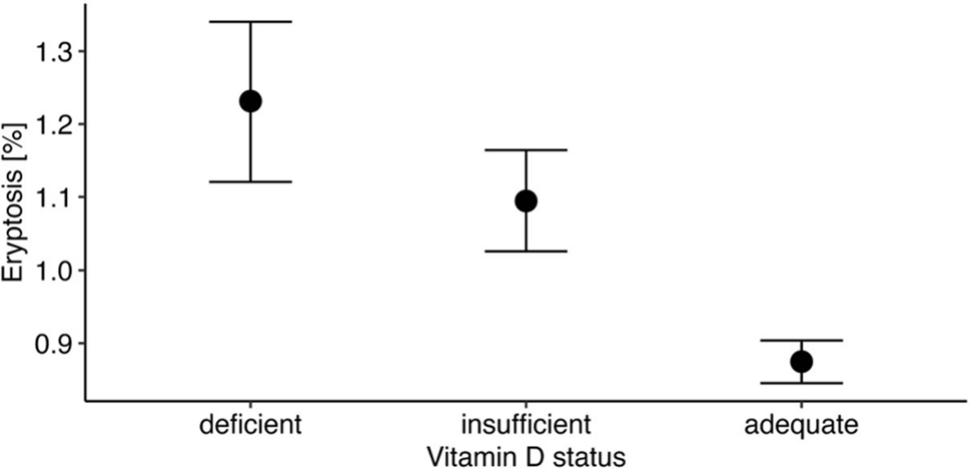
Eryptosis in study participants with vitamin D–deficient, vitamin D–insufficient, and vitamin D–adequate vitamin D status. The vitamin D status categories are based on plasma levels of 25(OH)D (deficient ≤ 30 nmol/l, insufficient 30 to < 50 nmol/l, and adequate ≥ 50 nmol/l) [30]. Points indicate posterior means and bars indicate 95% CI. The figure was created using the *R* programming language

Figure 4 illustrates that the eryptosis differed between the three vitamin D status groups, with the posterior means [95% CI] as follows: deficient 1.23% [1.12, 1.34], insufficient 1.09% [1.02, 1.16], and adequate 0.87% [0.85, 0.91]. Accordingly, vitamin D–deficient subjects having 25(OH)D levels ≤ 30 nmol/l were characterized by a 13% higher eryptosis rate than vitamin D–insufficient participants (Cohen’s *d* = 0.14, 95% CI [0.00, 0.26]) and a 41% higher eryptosis rate than vitamin D–adequate subjects (Cohen’s *d* = 0.36, 95% CI [0.24, 0.47]). Interestingly, individuals with insufficient vitamin D levels were also found to have a 25% higher eryptosis rate than those with an adequate vitamin D status (Cohen’s *d* = 0.22, 95% CI [0.14, 0.29]).

**Fig. 5:**
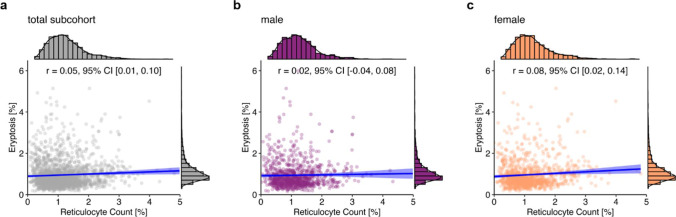
Correlation between reticulocyte count and eryptosis in the total NAKO subcohort (*n* = 2033; **a**), and grouped by sex (**b**: 962 males; **c**: 1,071 females). Histograms show the marginal distribution of the respective variable, lines indicate regression, and the blue ribbon around the regression line shows the uncertainty as 95% CI. The figure was created using the *R* programming language

Since higher eryptosis can be expected to stimulate erythropoiesis and in turn the number of circulating reticulocytes [31], an additional correlation analysis between reticulocyte count and eryptosis was conducted. In fact, it revealed a slight positive correlation in the total sample (*r* = 0.05, 95% CI [0.01, 0.10]; Fig. 5a). Additional analyses for men and women revealed no correlation between reticulocytes and eryptosis in men (*r* = 0.02, 95% CI [− 0.04, 0.08]; Fig. 5b), while there was a positive correlation between reticulocytes and eryptosis in women (*r* = 0.08, 95% CI [0.02, 0.14]; Fig. 5c).

**Fig. 6:**
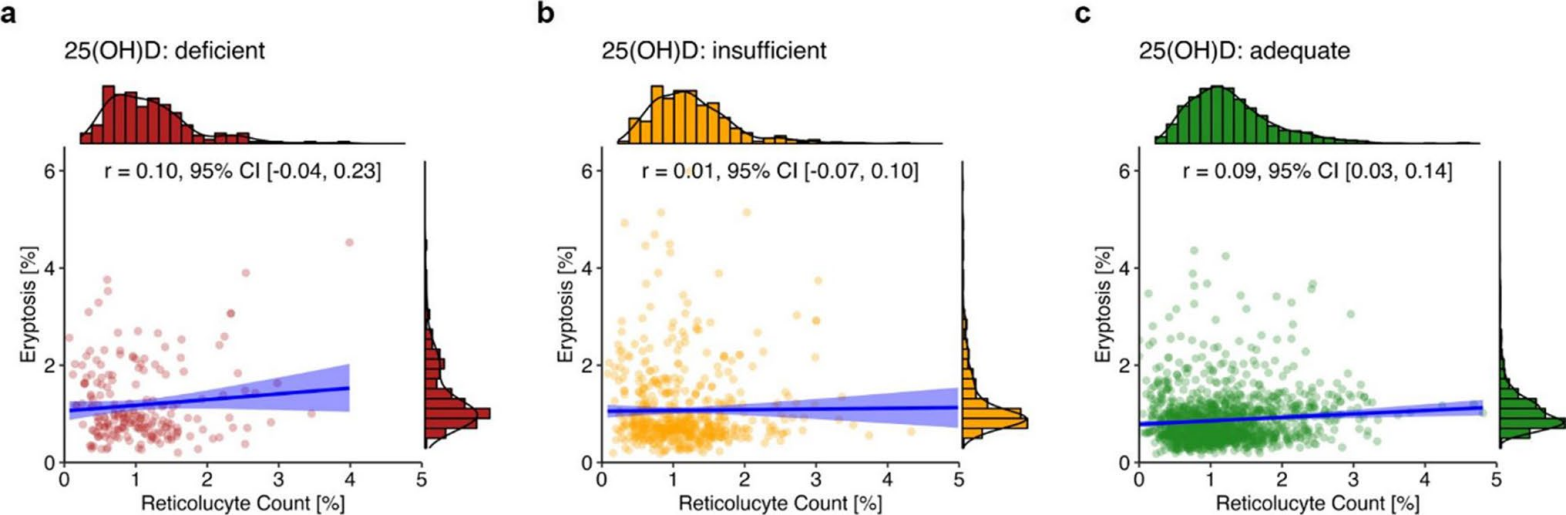
Correlation between reticulocyte count and eryptosis in the three categories of vitamin D status. Lines indicate regression, and the blue ribbon around the regression line shows the uncertainty as 95% CI. The figure was created using the *R* programming language

Correlation analysis for each vitamin D status group did not reveal any correlation between reticulocytes and eryptosis in the vitamin D–deficient (*r* = 0.10, 95% CI [− 0.04, 0.23]; Fig. 6a) and vitamin D–insufficient (*r* = 0.01, 95% CI [− 0.07, 0.10]; Fig. 6b) groups. In contrast, there was a small positive correlation between reticulocytes and eryptosis in the vitamin D–adequate group (*r* = 0.09, 95% CI [0.03, 0.14]; Fig. 6c).^1^

In a multiple regression analysis, there was a weak negative association between 25(OH)D and eryptosis after controlling for BMI, sex, age, and reticulocyte count (Table 2).

**Table 2:** Results of the multiple regression model for the outcome variable eryptosis. For each predictor variable, the posterior mean and 95% CI of the respective regression weight is reported

	Estimate	95% CI
25(OH)D (per 1 nmol/l)	− 0.004	[− 0.005, − 0.002]
Gender (female vs. male)	0.018	[− 0.038, 0.075]
BMI (per 1 kg/m^2^)	− 0.006	[− 0.011, 0.000]
Age (per year)	− 0.005	[− 0.007, − 0.003]
Reticulocyte count (per 1%)	0.077	[0.033, 0.121]

**Fig. 7:**
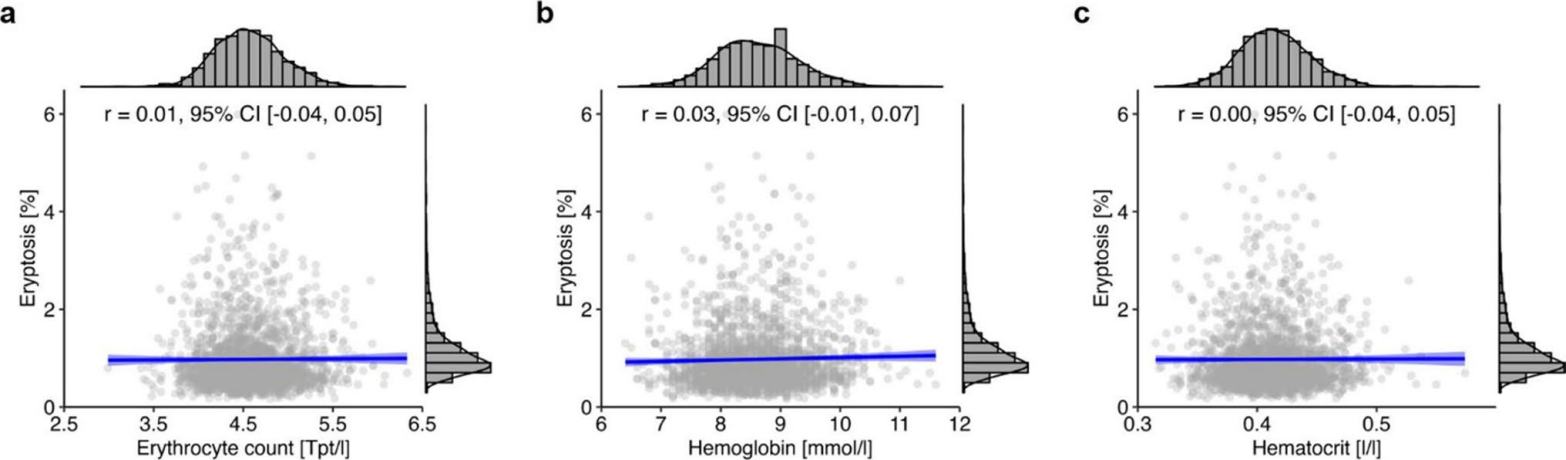
Relationship between eryptosis and hematological parameters in the total NAKO subcohort (*n* = 2045 for erythrocyte count and hemoglobin, *n* = 2044 for hematocrit). Histograms show the marginal distribution of the respective variable, lines indicate regression, and the blue ribbon around the regression line shows the uncertainty as 95% CI. The figure was created using the *R* programming language

To investigate whether the enhanced eryptosis is associated with lower erythrocyte counts, hemoglobin concentrations, or hematocrit, additional correlation analyses were performed. Interestingly, no correlations were observed between eryptosis and erythrocyte count (*r* = 0.01, 95% CI [− 0.04, 0.05]; Fig. 7a), hemoglobin (*r* = 0.03, 95% CI [− 0.01, 0.07]; Fig. 7b), or hematocrit (*r* = 0.00, 95% CI [− 0.04, 0.05]; Fig. 7c). However, there was a moderate positive correlation between eryptosis and thrombocyte count (*r* = 0.15, 95% CI [0.10, 0.19]), and a weak positive correlation between eryptosis and leukocyte count (*r* = 0.05, 95% CI [0.01, 0.09]) in the investigated NAKO subcohort.

**Fig. 8:**
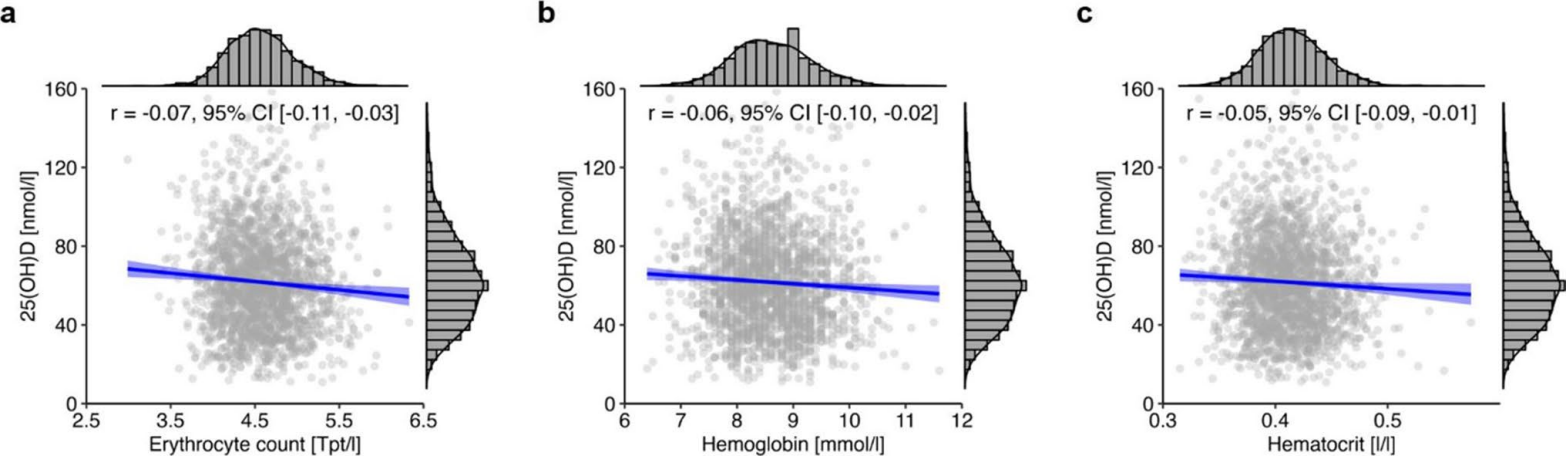
Correlation between 25(OH)D and hematological parameters in the total NAKO subcohort (*n* = 2045 for erythrocyte count and hemoglobin, *n* = 2044 for hematocrit). Histograms show the marginal distribution of the respective variable, lines indicate regression, and the blue ribbon around the regression line shows the uncertainty as 95% CI. The figure was created using the *R* programming language

Additionally, a weak inverse correlation was observed between 25(OH)D and erythrocyte count (*r* =  − 0.07, 95% CI [− 0.11, − 0.03]; Fig. 8a), hemoglobin (*r* =  − 0.06, 95% CI [− 0.10, − 0.02]; Fig. 8b), hematocrit (*r* =  − 0.05, 95% CI [− 0.09, − 0.01]; Fig. 8c), thrombocyte count (*r* =  − 0.05, 95% CI [− 0.09, − 0.01]), and leukocyte count (*r* =  − 0.08, 95% CI [− 0.13, − 0.04]).

## Discussion

This study was the first that investigated the association between vitamin D status, assessed by the measurement of plasma 25(OH)D and eryptosis in male and female subjects of the NAKO subcohort. Data from more than 2000 individuals showed a small negative correlation between circulating 25(OH)D and eryptosis. This means that a low vitamin D status was associated with higher eryptosis, although vitamin D could explain only 2% of the eryptosis variance. Overall, little association was found between vitamin D status and eryptosis, but when study participants were divided into three vitamin D status groups, individuals with vitamin D deficiency and vitamin D insufficiency had, on average, 41% and 25% higher eryptosis levels, respectively, than vitamin D–adequate subjects. Interestingly, cross-sectional analyses did not find relevant sex effects on eryptosis, but a negative correlation between age and eryptosis. In addition, a weak inverse correlation between BMI and eryptosis was found in men, but not in women. Interestingly, eryptosis was not associated with erythrocyte count, hemoglobin, and hematocrit, but positively associated with platelet count. In contrast, 25(OH)D was inversely correlated with blood cell counts, hemoglobin, and hematocrit, although this relationship was generally weak.

Erythrocytes in healthy subjects have a limited lifespan of approximately 3 months, which results from natural aging accompanied by, e.g., a disruption of cytoskeletal connections to the lipid bilayer, decreased membrane fluidity, or phospholipid scrambling [32]. Eryptosis is a phenomenon that is mechanistically distinct from the natural senescence of erythrocytes, and many substances or factors that are toxic to cells can cause this apoptosis-like suicidal cell death. Factors so far identified to stimulate eryptosis are e.g., oxidative stress [33], heavy metals such as copper [34] or cadmium [35], leukotriene C4 [36], and drugs [37] as well as clinical conditions including heart failure [38] or chronic kidney disease [39]. Eryptosis has also been linked to various infectious diseases, e.g., viral or parasitic infections [31]. Conversely, antioxidative factors such as vitamin E or glutathione have been identified as inhibitors of eryptosis [37]. However, the precise role of vitamin D in eryptosis remains enigmatic. Our study uncovered only a small association between vitamin D status and eryptosis, with individuals with vitamin D deficiency or insufficiency exhibiting a higher eryptosis rate than individuals with an adequate vitamin D status. Compared to mice fed a normal diet, mice on a high vitamin D diet tended to have lower eryptosis in freshly drawn blood [16]. In vitro exposure of erythrocytes from mice on a high vitamin D diet to eryptosis stimuli including hyperosmotic shock or glucose deprivation, however, resulted in significantly higher levels of eryptosis than exposure of erythrocytes from control mice [16]. However, it should be kept in mind that control mice can be assumed to have an adequate vitamin D status, whereas a high vitamin D diet may lead to unphysiologically high vitamin D levels. Our results are therefore not necessarily in contrast with this study. Klotho-deficient mice are another example of deranged vitamin D metabolism as these animals exhibit extremely high 1,25(OH)_2_D_3_ levels [40]. These animals show a significantly higher eryptosis rate than control mice, an effect that abrogated by low vitamin D diet feeding [41]. As our study population did not include individuals with abnormally high levels of vitamin D, the results of the two animal studies cannot be compared with our findings. The weak relationship between vitamin D and eryptosis may also explain why we did not find a deteriorated hematological status or anemia in subjects having a higher eryptosis rate.

Unexpectedly, individuals with better vitamin D status were more likely to have low erythrocyte count, hemoglobin, hematocrit, thrombocyte count, and leukocyte count. However, a negative association between vitamin D status and hematological parameters, including leukocyte count, was also found in other human studies [42–45], although other trials found no relationship [46]. So far, this phenomenon remains enigmatic. The observed inverse relationship between platelet count and 25(OH)D in the NAKO subcohort is consistent with the results from studies that found a higher vitamin D status associated with a lower platelet count [44, 45, 47]. However, antithrombotic effects of the vitamin D-vitamin D receptor axis have already been described [48] and indicate an important role of vitamin D in antithrombotic homeostasis.

Eryptosis is predominantly associated with pathophysiological processes since eryptotic red blood cells exposing PS on their outer membranes can interact with surface receptors of endothelial cells and platelets [49] thereby adhering to the inner blood vessel walls and platelets which may subsequently impede the local circulation or increase thrombosis risk [49–51]. The clinical significance of enhanced eryptosis in humans is not known, but data from mice show that accelerated eryptosis can contribute to anemia and splenomegaly [52] as well as microangio- and coagulopathies [53]. Therefore, a higher eryptosis associated with vitamin D deficiency could impact the hematologic and cardiovascular system, although the current study did not find any negative impact of the increased eryptosis on hematologic status. It is possible that the higher risk of anemia associated with a low vitamin D status [8–12] and the inverse correlations between 25(OH)D levels and red blood cell count, hemoglobin concentration, and mean corpuscular hemoglobin observed in the *German Health Interview Survey for Children and Adolescents* [42] are not caused by enhanced eryptosis. Whether higher eryptosis observed in vitamin D–deficient and vitamin D–insufficient individuals in comparison to vitamin D–adequate subjects constitutes a relevant risk for cardiovascular events remains unclear. Cardiovascular complications in vitamin D–deficient patients are infrequently reported, and data do not support a major effect of vitamin D supplementation in the general population or in the clinical setting to reduce CVD risk [54]. The current findings which show a robust, but overall weak, correlation between vitamin D and eryptosis fit very well into this picture.

## Strengths and limitations 

To our knowledge, this is the first study in humans that investigated the relationship between vitamin D status and eryptosis and analyzed eryptosis in the larger population group. The measurement of 25(OH)D and eryptosis was conducted in more than 2000 participants, to obtain sufficient data for a possible correlation between these two variables. A limitation of this study was that the health status of the subjects, including, for example, infectious diseases, on eryptosis was not considered in the analysis.

To conclude, the observational data indicate a weak, but clear association between a low vitamin D status and an increased eryptosis. Whether the observed differences in eryptosis between vitamin D–deficient and vitamin D–adequate participants are relevant in terms of an increased risk for anemia or cardiovascular events could not be evaluated in the studied sample. In addition, the current study showed that age and BMI in males are other factors associated with eryptosis in humans.

## Data Availability

The datasets generated during and/or analyzed during the current study are not publicly available but are available from the corresponding author on reasonable request.

## Footnotes

1 While the point estimates (posterior mean) for the regression coefficient *r* are roughly equal, the credible intervals reveal that the posterior distribution for the correlation coefficient in the vitamin D–deficient group is broader than for the vitamin D–adequate group.
